# P-59. Tdap Vaccination Coverage During Pregnancy, 2016 Through 2023

**DOI:** 10.1093/ofid/ofae631.266

**Published:** 2025-01-29

**Authors:** Stephanie Irving, Bradley Crane, Eric Weintraub, Suchita A Patel, Hilda Razzaghi, Edward Belongia, Matthew F Daley, Darios Gethaun, Sungching C Glenn, Simon Hambidge, Lisa Jackson, Elyse Kharbanda, Nicola P Klein, Ousseny Zerbo, Allison L Naleway

**Affiliations:** Kaiser Permanente Center for Health Research, Portland, Oregon; Kaiser Permanente Center for Health Research, Portland, Oregon; Centers for Disease Control and Prevention, Atlanta, GA; Centers for Disease Control and Prevention, Atlanta, GA; CDC, Atlanta, Georgia; Marshfield Clinic Research Institute, Marshfield, WI; Kaiser Permanente Colorado, Aurora, Colorado; 5Kaiser Permanente Department of Research and Evaluation, Pasadena, California, Pasadena, California; Kaiser Permanente Southern California, Pasadena, California; Denver Health, Denver, CO; Kaiser Permanente Washington Health Research Institute, Seattle, WA; HealthPartners Institute, Minneapolis, Minnesota; Division of Research Kaiser Permanente Vaccine Study Center, Oakland, California; Division of Research Kaiser Permanente Vaccine Study Center, Oakland, California; Kaiser Permanente Center for Health Research, Portland, Oregon

## Abstract

**Background:**

The Advisory Committee on Immunization Practices recommends tetanus-diphtheria-acellular pertussis (Tdap) vaccination during every pregnancy, preferably between gestational weeks 27 and 36. Estimates of Tdap vaccination coverage among pregnant people for the period overlapping the COVID-10 pandemic are limited. Our objective was to assess Tdap vaccination coverage during pregnancy within the Vaccine Safety Datalink (VSD) before and during the COVID-19 pandemic, to examine the potential impact of the pandemic on Tdap coverage.

Tdap vaccination coverage during pregnancy, overall and by age group – 2016 through 2023, Vaccine Safety Datalink

**Figure:**
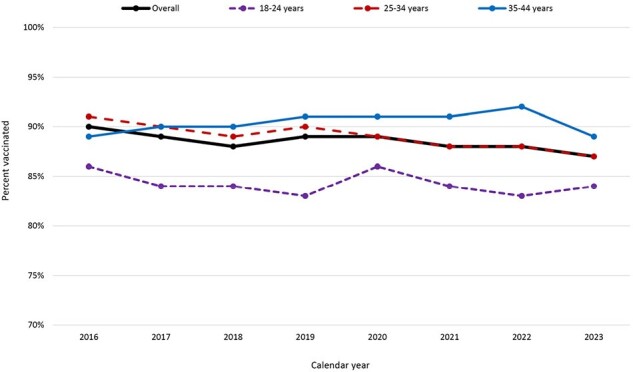

Tdap vaccination coverage among pregnant people ages 12--54 years, overall and stratified by age group. Ages 12--17 and 45--54 years have been excluded from stratified estimates due to low numbers, but are included in the overall estimate. Eligible pregnancies had a live birth of at least 26 weeks’ gestation during the study period and were continuously enrolled at their Vaccine Safety Datalink site for the duration of their pregnancy. Age was determined as of pregnancy start. Data available and presented here are limited to one VSD site; data from eight other VSD sites are being collected.

**Methods:**

We examined Tdap vaccination coverage among pregnant people ages 12—54 years with a live birth of ≥ 26 weeks’ gestation from January 2016 through December 2023; pregnancies were identified using a validated algorithm. We captured Tdap, influenza, COVID-19, and respiratory syncytial virus (RSV) vaccination during the pregnancy using electronic medical records linked to immunization registries. We assessed annual crude Tdap vaccination coverage and demographics associated with Tdap vaccination. Among people who received Tdap, we examined gestational age at Tdap receipt and described uptake of other vaccines recommended during pregnancy (influenza, COVID-19, RSV). Data available and presented here are limited to one VSD site; data from eight other VSD sites are being collected.

Tdap vaccination coverage during pregnancy, by race and ethnicity – 2016 through 2023, Vaccine Safety Datalink

**Figure:**
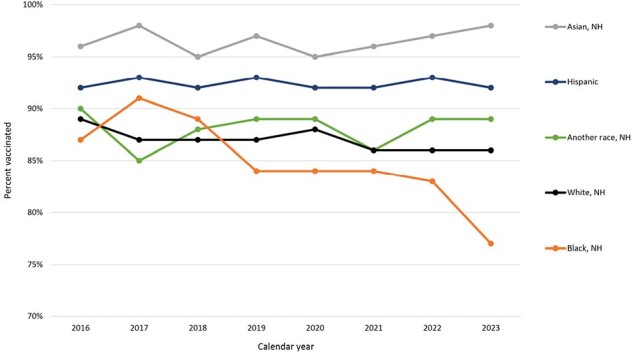

Tdap vaccination coverage among pregnant people ages 12-54 years, stratified by race and ethnicity. Eligible pregnancies had a live birth of at least 26 weeks’ gestation during the study period and were continuously enrolled at their Vaccine Safety Datalink site for the duration of their pregnancy. Race and ethnicity were assessed using electronic health record data. The following race and ethnicity categories were combined into “Another race, NH” due to low numbers: non-Hispanic (NH) multiple race [3.7%], NH Native Hawaiian or Pacific Islander [0.8%], NH Middle Eastern or North African [0.7%], NH American Indian or Alaskan Native [0.4%]. Data available and presented here are limited to one VSD site; data from eight other VSD sites are being collected.

**Results:**

An average of 4,143 births were included in each of the eight calendar years. The population was, on average, 64% non-Hispanic (NH) White, 13% Hispanic, 9% NH Asian, 6% NH multiracial, 5% unknown race and ethnicity, and 4% NH Black; 62% of pregnant people were 25—34 years old. Crude Tdap vaccination coverage was 90% among those with births in 2016 and 87% among those with births in 2023. Coverage was consistently lowest among those aged 18—24 (Figure 1). In all years, NH Asian pregnant people had the highest Tdap coverage; coverage was lowest among NH Black pregnant people in six of eight years, with a sharp drop in coverage in 2023 (Figure 2). Patterns of vaccination among pregnant people who received Tdap are shown in Figure 3.

Patterns of Tdap, influenza, COVID-19, and RSV vaccine uptake during pregnancy among those who received Tdap during pregnancy – 2016 through 2023, Vaccine Safety Datalink

**Figure:**
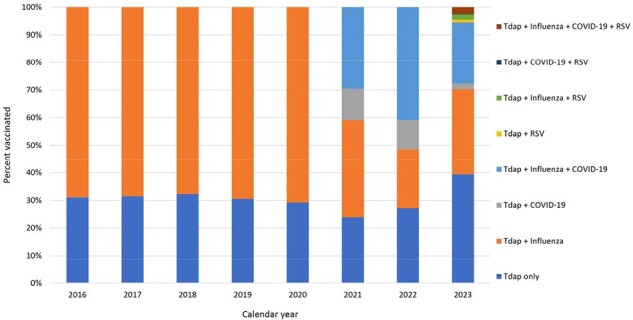

Tdap, influenza, COVID-19, and RSV vaccination during pregnancy among individuals who received Tdap during pregnancy. Eligible pregnancies had a live birth of at least 26 weeks’ gestation during the study period and were continuously enrolled at their Vaccine Safety Datalink site for the duration of their pregnancy. Data available and presented here are limited to one VSD site; data from eight other VSD sites are being collected.

**Conclusion:**

Between 2016 and 2023, 87—90% of pregnant people with live births received the Tdap vaccine each year. However, we identified consistent disparities in coverage by age, race, and ethnicity; racial disparities widened in later years.

**Disclosures:**

**Darios Gethaun, MD, PhD, MPH**, HOLOGIC Inc.: Grant/Research Support|Johnson & Johnson: Grant/Research Support **Nicola P. Klein, MD, PhD**, CSL Seqirus: Grant/Research Support|GlaxoSmithKline: Grant/Research Support|Merck: Grant/Research Support|Pfizer: Grant/Research Support|Sanofi Pasteur: Grant/Research Support

